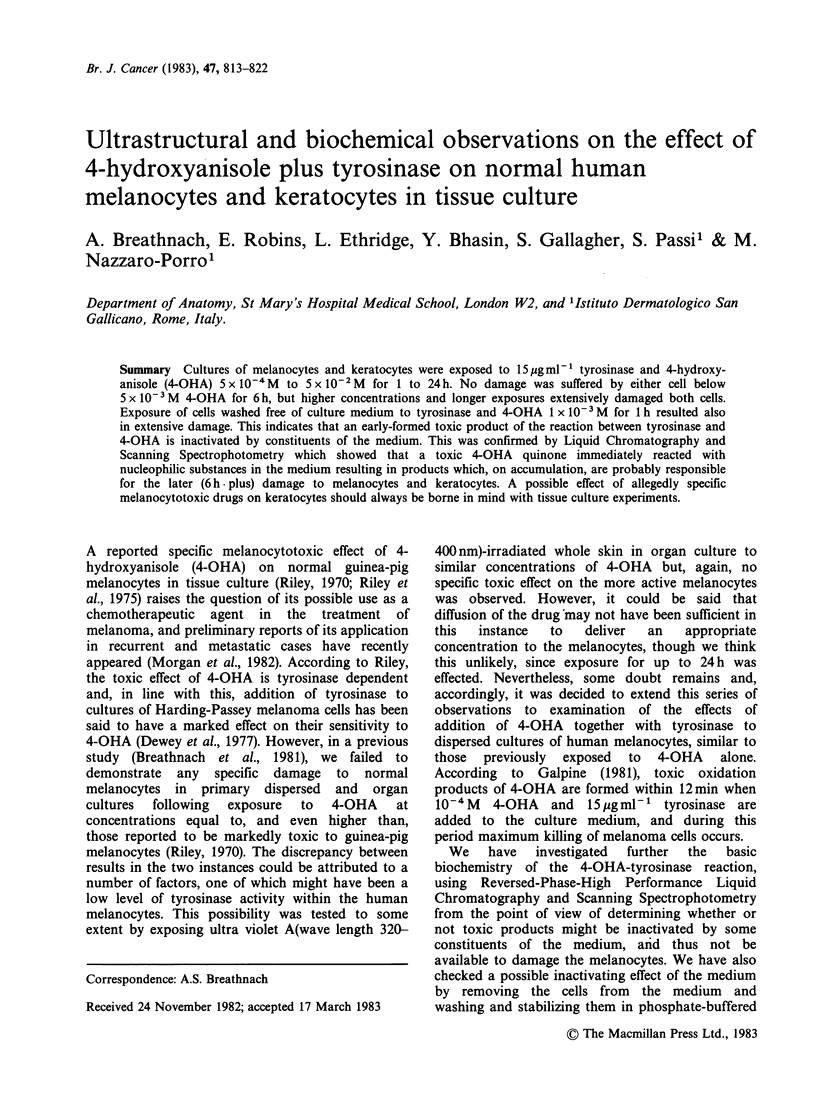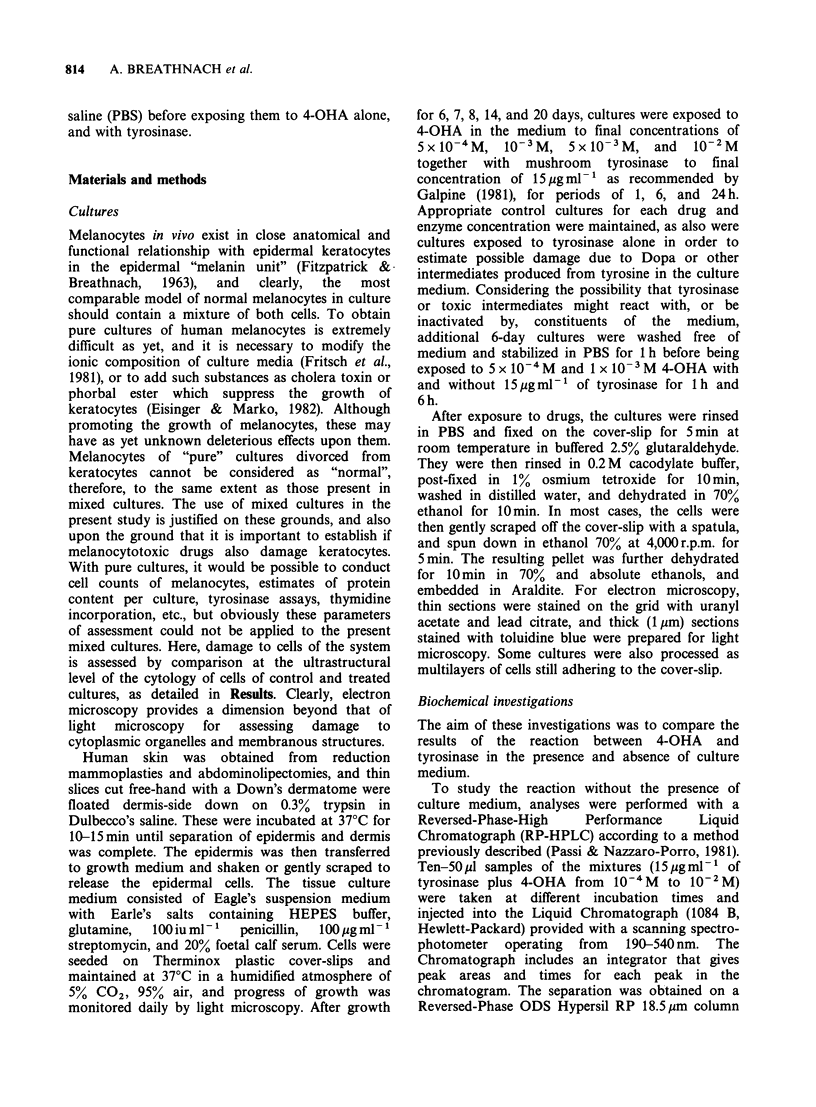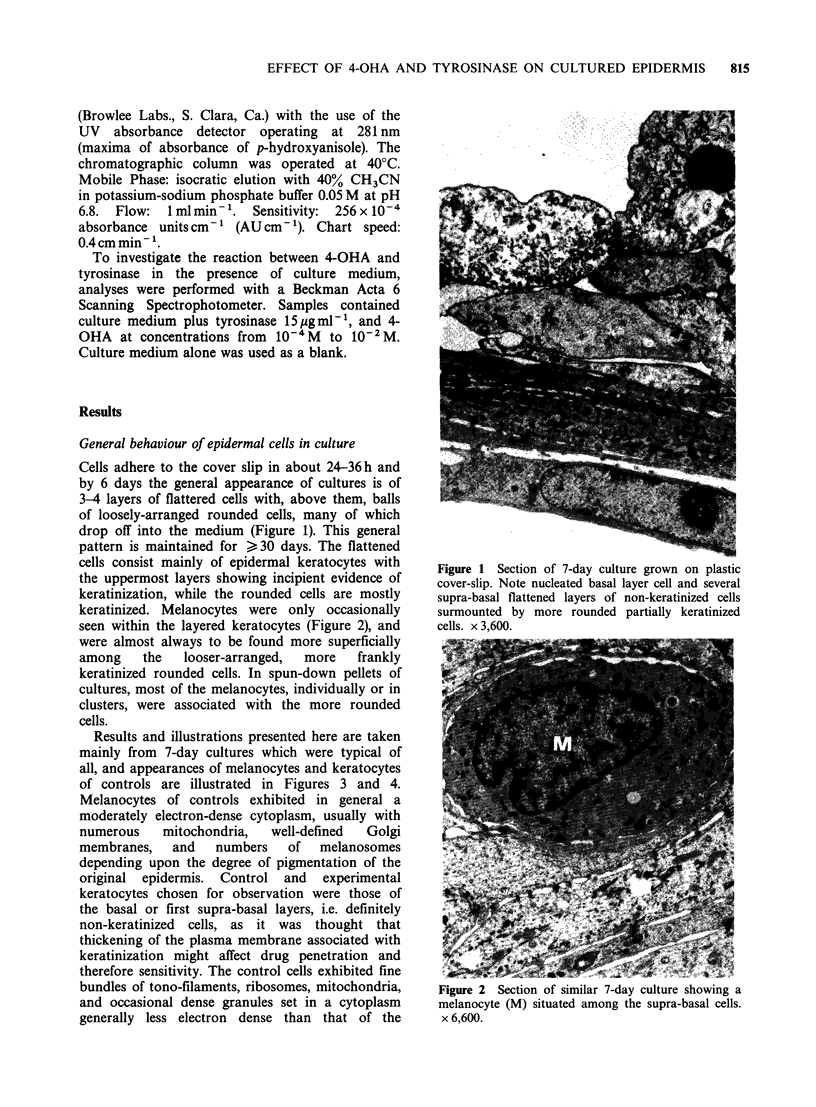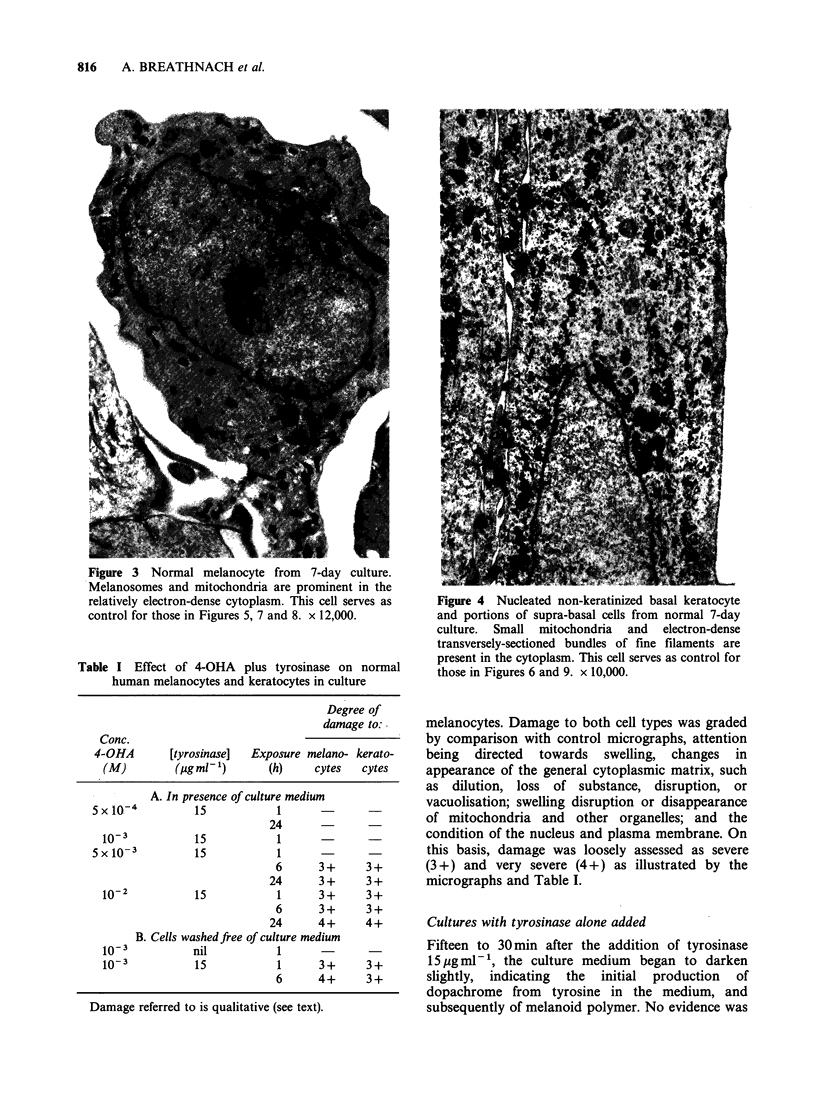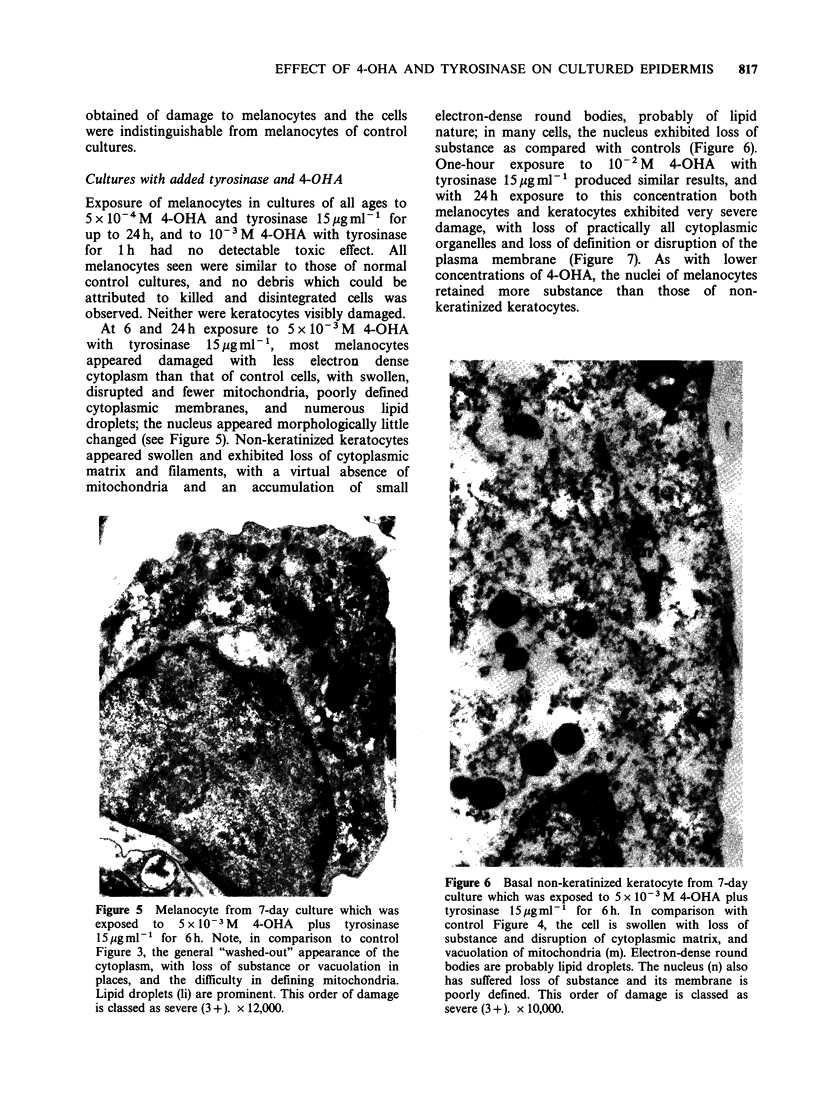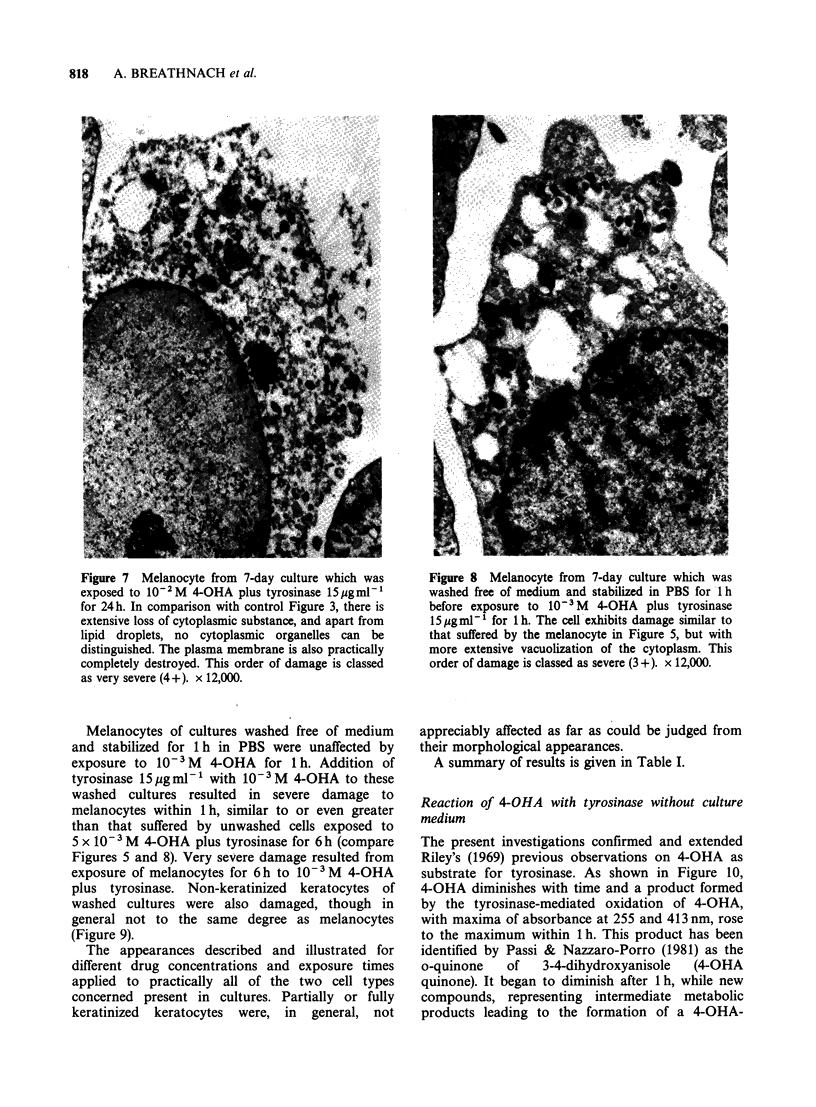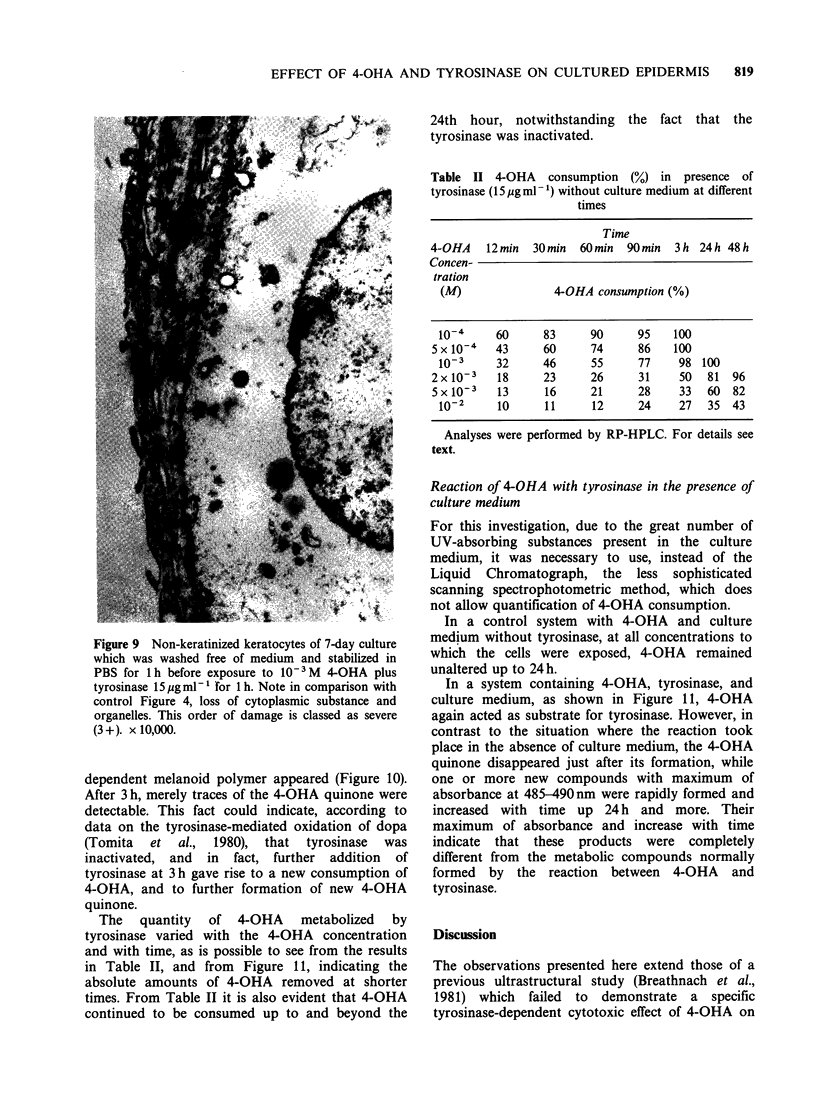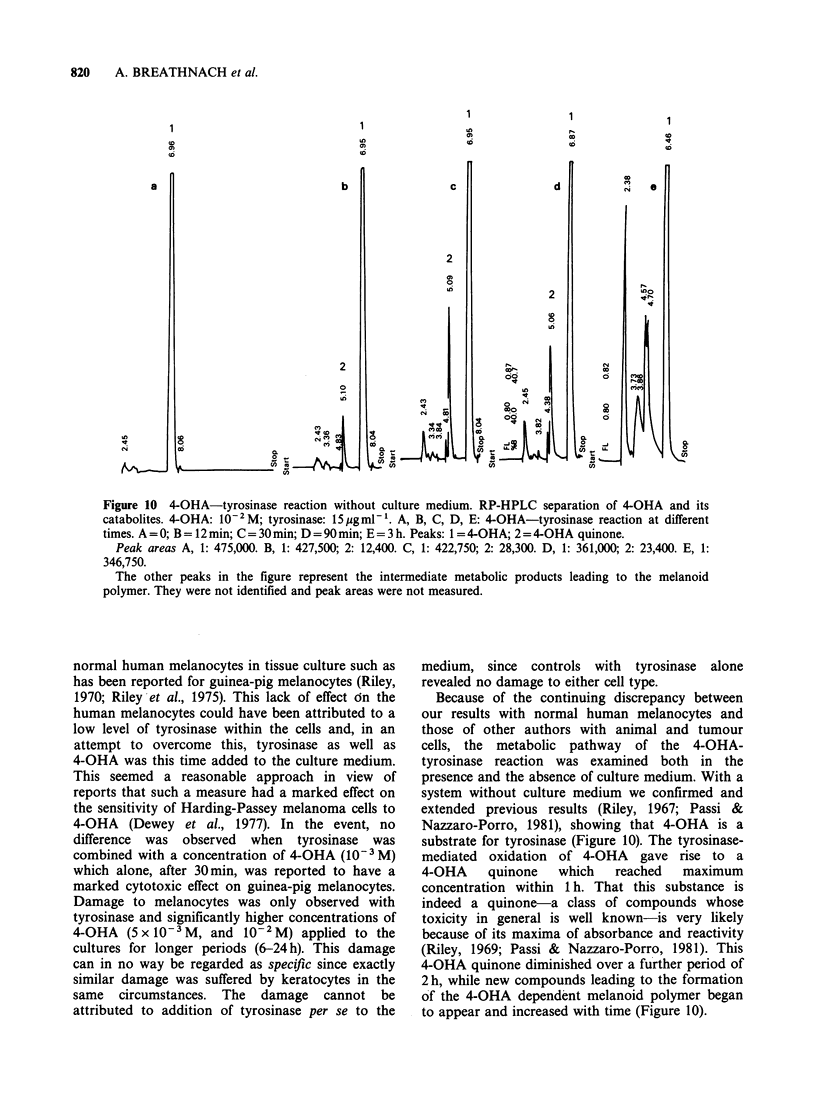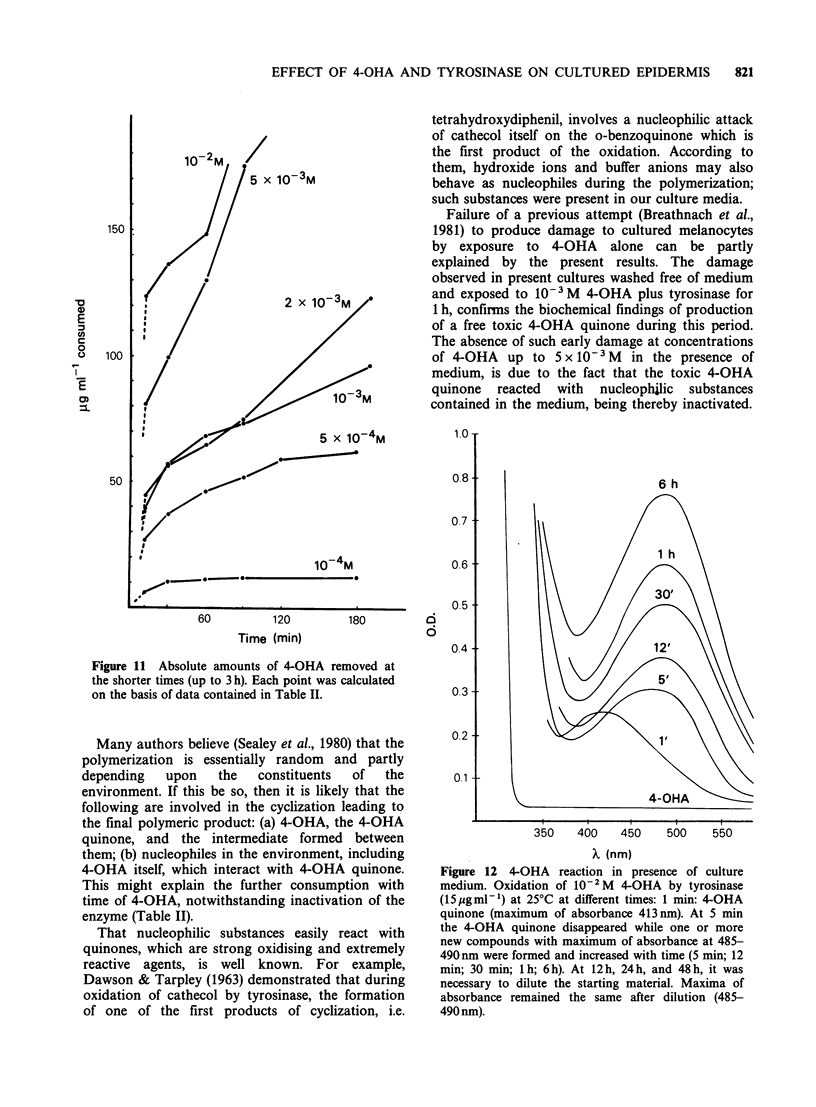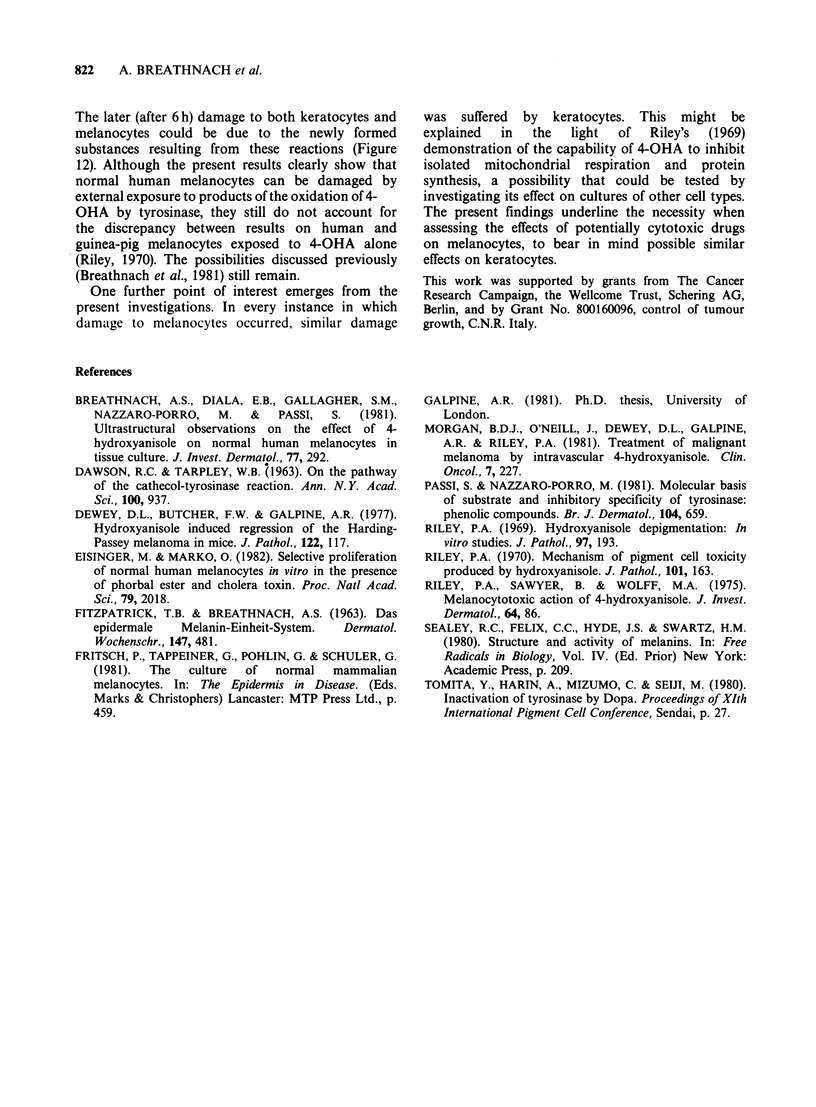# Ultrastructural and biochemical observations on the effect of 4-hydroxyanisole plus tyrosinase on normal human melanocytes and keratocytes in tissue culture.

**DOI:** 10.1038/bjc.1983.136

**Published:** 1983-06

**Authors:** A. Breathnach, E. Robins, L. Ethridge, Y. Bhasin, S. Gallagher, S. Passi, M. Nazzaro-Porro

## Abstract

**Images:**


*Br. J. Cancer* (1983), **47**, 813–822

### Ultrastructural and biochemical observations on the effect of 4-hydroxyanisole plus tyrosinase on normal human melanocytes and keratocytes in tissue culture

A. Breathnach, E. Robins, L. Ethridge, Y. Bhasin, S. Gallagher, S. Passi[1] & M. Nazzaro-Porro[1]

*Department of Anatomy, St Mary's Hospital Medical School, London W2, and [1]Istituto Dermatologico San Gallicano, Rome, Italy.*

**Summary** Cultures of melanocytes and keratocytes were exposed to $15\,\mu g\,ml^{-1}$ tyrosinase and 4-hydroxyanisole (4-OHA) $5\times10^{-4}$M to $5\times10^{-2}$M for 1 to 24h. No damage was suffered by either cell below $5\times10^{-3}$M 4-OHA for 6h, but higher concentrations and longer exposures extensively damaged both cells. Exposure of cells washed free of culture medium to tyrosinase and 4-OHA $1\times10^{-3}$M for 1h resulted also in extensive damage. This indicates that an early-formed toxic product of the reaction between tyrosinase and 4-OHA is inactivated by constituents of the medium. This was confirmed by Liquid Chromatography and Scanning Spectrophotometry which showed that a toxic 4-OHA quinone immediately reacted with nucleophilic substances in the medium resulting in products which, on accumulation, are probably responsible for the later (6h · plus) damage to melanocytes and keratocytes. A possible effect of allegedly specific melanocytotoxic drugs on keratocytes should always be borne in mind with tissue culture experiments.

A reported specific melanocytotoxic effect of 4-hydroxyanisole (4-OHA) on normal guinea-pig melanocytes in tissue culture (Riley, 1970; Riley *et al.*, 1975) raises the question of its possible use as a chemotherapeutic agent in the treatment of melanoma, and preliminary reports of its application in recurrent and metastatic cases have recently appeared (Morgan *et al.*, 1982). According to Riley, the toxic effect of 4-OHA is tyrosinase dependent and, in line with this, addition of tyrosinase to cultures of Harding-Passey melanoma cells has been said to have a marked effect on their sensitivity to 4-OHA (Dewey *et al.*, 1977). However, in a previous study (Breathnach *et al.*, 1981), we failed to demonstrate any specific damage to normal melanocytes in primary dispersed and organ cultures following exposure to 4-OHA at concentrations equal to, and even higher than, those reported to be markedly toxic to guinea-pig melanocytes (Riley, 1970). The discrepancy between results in the two instances could be attributed to a number of factors, one of which might have been a low level of tyrosinase activity within the human melanocytes. This possibility was tested to some extent by exposing ultra violet A(wave length 320–400nm)-irradiated whole skin in organ culture to similar concentrations of 4-OHA but, again, no specific toxic effect on the more active melanocytes was observed. However, it could be said that diffusion of the drug may not have been sufficient in this instance to deliver an appropriate concentration to the melanocytes, though we think this unlikely, since exposure for up to 24h was effected. Nevertheless, some doubt remains and, accordingly, it was decided to extend this series of observations to examination of the effects of addition of 4-OHA together with tyrosinase to dispersed cultures of human melanocytes, similar to those previously exposed to 4-OHA alone. According to Galpine (1981), toxic oxidation products of 4-OHA are formed within 12min when $10^{-4}$M 4-OHA and $15\,\mu g\,ml^{-1}$ tyrosinase are added to the culture medium, and during this period maximum killing of melanoma cells occurs.

We have investigated further the basic biochemistry of the 4-OHA-tyrosinase reaction, using Reversed-Phase-High Performance Liquid Chromatography and Scanning Spectrophotometry from the point of view of determining whether or not toxic products might be inactivated by some constituents of the medium, and thus not be available to damage the melanocytes. We have also checked a possible inactivating effect of the medium by removing the cells from the medium and washing and stabilizing them in phosphate-buffered

Correspondence: A.S. Breathnach

Received 24 November 1982; accepted 17 March 1983

© The Macmillan Press Ltd., 1983

saline (PBS) before exposing them to 4-OHA alone, and with tyrosinase.

### Materials and methods

### *Cultures*

Melanocytes *in vivo* exist in close anatomical and functional relationship with epidermal keratocytes in the epidermal "melanin unit" (Fitzpatrick & Breathnach, 1963), and clearly, the most comparable model of normal melanocytes in culture should contain a mixture of both cells. To obtain pure cultures of human melanocytes is extremely difficult as yet, and it is necessary to modify the ionic composition of culture media (Fritsch *et al.*, 1981), or to add such substances as cholera toxin or phorbal ester which suppress the growth of keratocytes (Eisinger & Marko, 1982). Although promoting the growth of melanocytes, these may have as yet unknown deleterious effects upon them. Melanocytes of "pure" cultures divorced from keratocytes cannot be considered as "normal", therefore, to the same extent as those present in mixed cultures. The use of mixed cultures in the present study is justified on these grounds, and also upon the ground that it is important to establish if melanocytotoxic drugs also damage keratocytes. With pure cultures, it would be possible to conduct cell counts of melanocytes, estimates of protein content per culture, tyrosinase assays, thymidine incorporation, etc., but obviously these parameters of assessment could not be applied to the present mixed cultures. Here, damage to cells of the system is assessed by comparison at the ultrastructural level of the cytology of cells of control and treated cultures, as detailed in **Results**. Clearly, electron microscopy provides a dimension beyond that of light microscopy for assessing damage to cytoplasmic organelles and membranous structures.

Human skin was obtained from reduction mammoplasties and abdominolipectomies, and thin slices cut free-hand with a Down's dermatome were floated dermis-side down on 0.3% trypsin in Dulbecco's saline. These were incubated at 37°C for 10–15 min until separation of epidermis and dermis was complete. The epidermis was then transferred to growth medium and shaken or gently scraped to release the epidermal cells. The tissue culture medium consisted of Eagle's suspension medium with Earle's salts containing HEPES buffer, glutamine, 100 iu $ml^{-1}$ penicillin, 100 $\mu g\,ml^{-1}$ streptomycin, and 20% foetal calf serum. Cells were seeded on Therminox plastic cover-slips and maintained at 37°C in a humidified atmosphere of 5% $CO_2$, 95% air, and progress of growth was monitored daily by light microscopy. After growth for 6, 7, 8, 14, and 20 days, cultures were exposed to 4-OHA in the medium to final concentrations of $5\times10^{-4}$ M, $10^{-3}$ M, $5\times10^{-3}$ M, and $10^{-2}$ M together with mushroom tyrosinase to final concentration of 15 $\mu g\,ml^{-1}$ as recommended by Galpine (1981), for periods of 1, 6, and 24 h. Appropriate control cultures for each drug and enzyme concentration were maintained, as also were cultures exposed to tyrosinase alone in order to estimate possible damage due to Dopa or other intermediates produced from tyrosine in the culture medium. Considering the possibility that tyrosinase or toxic intermediates might react with, or be inactivated by, constituents of the medium, additional 6-day cultures were washed free of medium and stabilized in PBS for 1 h before being exposed to $5\times10^{-4}$ M and $1\times10^{-3}$ M 4-OHA with and without 15 $\mu g\,ml^{-1}$ of tyrosinase for 1 h and 6 h.

After exposure to drugs, the cultures were rinsed in PBS and fixed on the cover-slip for 5 min at room temperature in buffered 2.5% glutaraldehyde. They were then rinsed in 0.2 M cacodylate buffer, post-fixed in 1% osmium tetroxide for 10 min, washed in distilled water, and dehydrated in 70% ethanol for 10 min. In most cases, the cells were then gently scraped off the cover-slip with a spatula, and spun down in ethanol 70% at 4,000 r.p.m. for 5 min. The resulting pellet was further dehydrated for 10 min in 70% and absolute ethanols, and embedded in Araldite. For electron microscopy, thin sections were stained on the grid with uranyl acetate and lead citrate, and thick (1 $\mu$m) sections stained with toluidine blue were prepared for light microscopy. Some cultures were also processed as multilayers of cells still adhering to the cover-slip.

### *Biochemical investigations*

The aim of these investigations was to compare the results of the reaction between 4-OHA and tyrosinase in the presence and absence of culture medium.

To study the reaction without the presence of culture medium, analyses were performed with a Reversed-Phase-High Performance Liquid Chromatograph (RP-HPLC) according to a method previously described (Passi & Nazzaro-Porro, 1981). Ten–50 $\mu$l samples of the mixtures (15 $\mu g\,ml^{-1}$ of tyrosinase plus 4-OHA from $10^{-4}$ M to $10^{-2}$ M) were taken at different incubation times and injected into the Liquid Chromatograph (1084 B, Hewlett-Packard) provided with a scanning spectrophotometer operating from 190–540 nm. The Chromatograph includes an integrator that gives peak areas and times for each peak in the chromatogram. The separation was obtained on a Reversed-Phase ODS Hypersil RP 18.5 $\mu$m column

(Browlee Labs., S. Clara, Ca.) with the use of the UV absorbance detector operating at 281 nm (maxima of absorbance of *p*-hydroxyanisole). The chromatographic column was operated at 40°C. Mobile Phase: isocratic elution with 40% $CH_3CN$ in potassium-sodium phosphate buffer 0.05 M at pH 6.8. Flow: 1 ml $min^{-1}$. Sensitivity: $256 \times 10^{-4}$ absorbance units $cm^{-1}$ (AU $cm^{-1}$). Chart speed: 0.4 cm $min^{-1}$.

To investigate the reaction between 4-OHA and tyrosinase in the presence of culture medium, analyses were performed with a Beckman Acta 6 Scanning Spectrophotometer. Samples contained culture medium plus tyrosinase 15 $\mu g\,ml^{-1}$, and 4-OHA at concentrations from $10^{-4}$ M to $10^{-2}$ M. Culture medium alone was used as a blank.

### Results

### *General behaviour of epidermal cells in culture*

Cells adhere to the cover slip in about 24–36 h and by 6 days the general appearance of cultures is of 3–4 layers of flattered cells with, above them, balls of loosely-arranged rounded cells, many of which drop off into the medium (Figure 1). This general pattern is maintained for $\geqslant$30 days. The flattened cells consist mainly of epidermal keratocytes with the uppermost layers showing incipient evidence of keratinization, while the rounded cells are mostly keratinized. Melanocytes were only occasionally seen within the layered keratocytes (Figure 2), and were almost always to be found more superficially among the looser-arranged, more frankly keratinized rounded cells. In spun-down pellets of cultures, most of the melanocytes, individually or in clusters, were associated with the more rounded cells.

Results and illustrations presented here are taken mainly from 7-day cultures which were typical of all, and appearances of melanocytes and keratocytes of controls are illustrated in Figures 3 and 4. Melanocytes of controls exhibited in general a moderately electron-dense cytoplasm, usually with numerous mitochondria, well-defined Golgi membranes, and numbers of melanosomes depending upon the degree of pigmentation of the original epidermis. Control and experimental keratocytes chosen for observation were those of the basal or first supra-basal layers, i.e. definitely non-keratinized cells, as it was thought that thickening of the plasma membrane associated with keratinization might affect drug penetration and therefore sensitivity. The control cells exhibited fine bundles of tono-filaments, ribosomes, mitochondria, and occasional dense granules set in a cytoplasm generally less electron dense than that of the

**Figure 1** Section of 7-day culture grown on plastic cover-slip. Note nucleated basal layer cell and several supra-basal flattened layers of non-keratinized cells surmounted by more rounded partially keratinized cells. ×3,600.

**Figure 2** Section of similar 7-day culture showing a melanocyte (M) situated among the supra-basal cells. ×6,600.

**Figure 3** Normal melanocyte from 7-day culture. Melanosomes and mitochondria are prominent in the relatively electron-dense cytoplasm. This cell serves as control for those in Figures 5, 7 and 8. ×12,000.

**Figure 4** Nucleated non-keratinized basal keratocyte and portions of supra-basal cells from normal 7-day culture. Small mitochondria and electron-dense transversely-sectioned bundles of fine filaments are present in the cytoplasm. This cell serves as control for those in Figures 6 and 9. ×10,000.

**Table I** Effect of 4-OHA plus tyrosinase on normal human melanocytes and keratocytes in culture

| Conc. 4-OHA (M) | [tyrosinase] ($\mu g\,ml^{-1}$) | Exposure (h) | Degree of damage to: melano-cytes | Degree of damage to: kerato-cytes |
|---|---|---|---|---|
| *A. In presence of culture medium* | | | | |
| $5\times10^{-4}$ | 15 | 1 | — | — |
| | | 24 | — | — |
| $10^{-3}$ | 15 | 1 | — | — |
| $5\times10^{-3}$ | 15 | 1 | — | — |
| | | 6 | 3+ | 3+ |
| | | 24 | 3+ | 3+ |
| $10^{-2}$ | 15 | 1 | 3+ | 3+ |
| | | 6 | 3+ | 3+ |
| | | 24 | 4+ | 4+ |
| *B. Cells washed free of culture medium* | | | | |
| $10^{-3}$ | nil | 1 | — | — |
| $10^{-3}$ | 15 | 1 | 3+ | 3+ |
| | | 6 | 4+ | 3+ |

Damage referred to is qualitative (see text).

melanocytes. Damage to both cell types was graded by comparison with control micrographs, attention being directed towards swelling, changes in appearance of the general cytoplasmic matrix, such as dilution, loss of substance, disruption, or vacuolisation; swelling disruption or disappearance of mitochondria and other organelles; and the condition of the nucleus and plasma membrane. On this basis, damage was loosely assessed as severe (3+) and very severe (4+) as illustrated by the micrographs and Table I.

### *Cultures with tyrosinase alone added*

Fifteen to 30 min after the addition of tyrosinase $15\,\mu g\,ml^{-1}$, the culture medium began to darken slightly, indicating the initial production of dopachrome from tyrosine in the medium, and subsequently of melanoid polymer. No evidence was obtained of damage to melanocytes and the cells were indistinguishable from melanocytes of control cultures.

*Cultures with added tyrosinase and 4-OHA*

Exposure of melanocytes in cultures of all ages to $5 \times 10^{-4}$M 4-OHA and tyrosinase $15 \mu g\,ml^{-1}$ for up to 24h, and to $10^{-3}$M 4-OHA with tyrosinase for 1h had no detectable toxic effect. All melanocytes seen were similar to those of normal control cultures, and no debris which could be attributed to killed and disintegrated cells was observed. Neither were keratocytes visibly damaged.

At 6 and 24h exposure to $5 \times 10^{-3}$M 4-OHA with tyrosinase $15 \mu g\,ml^{-1}$, most melanocytes appeared damaged with less electron dense cytoplasm than that of control cells, with swollen, disrupted and fewer mitochondria, poorly defined cytoplasmic membranes, and numerous lipid droplets; the nucleus appeared morphologically little changed (see Figure 5). Non-keratinized keratocytes appeared swollen and exhibited loss of cytoplasmic matrix and filaments, with a virtual absence of mitochondria and an accumulation of small electron-dense round bodies, probably of lipid nature; in many cells, the nucleus exhibited loss of substance as compared with controls (Figure 6). One-hour exposure to $10^{-2}$M 4-OHA with tyrosinase $15 \mu g\,ml^{-1}$ produced similar results, and with 24h exposure to this concentration both melanocytes and keratocytes exhibited very severe damage, with loss of practically all cytoplasmic organelles and loss of definition or disruption of the plasma membrane (Figure 7). As with lower concentrations of 4-OHA, the nuclei of melanocytes retained more substance than those of non-keratinized keratocytes.

**Figure 5** Melanocyte from 7-day culture which was exposed to $5 \times 10^{-3}$M 4-OHA plus tyrosinase $15 \mu g\,ml^{-1}$ for 6h. Note, in comparison to control Figure 3, the general "washed-out" appearance of the cytoplasm, with loss of substance or vacuolation in places, and the difficulty in defining mitochondria. Lipid droplets (li) are prominent. This order of damage is classed as severe (3+). ×12,000.

**Figure 6** Basal non-keratinized keratocyte from 7-day culture which was exposed to $5 \times 10^{-3}$M 4-OHA plus tyrosinase $15 \mu g\,ml^{-1}$ for 6h. In comparison with control Figure 4, the cell is swollen with loss of substance and disruption of cytoplasmic matrix, and vacuolation of mitochondria (m). Electron-dense round bodies are probably lipid droplets. The nucleus (n) also has suffered loss of substance and its membrane is poorly defined. This order of damage is classed as severe (3+). ×10,000.

**Figure 7** Melanocyte from 7-day culture which was exposed to $10^{-2}$ M 4-OHA plus tyrosinase 15 $\mu g\, ml^{-1}$ for 24 h. In comparison with control Figure 3, there is extensive loss of cytoplasmic substance, and apart from lipid droplets, no cytoplasmic organelles can be distinguished. The plasma membrane is also practically completely destroyed. This order of damage is classed as very severe (4+). ×12,000.

**Figure 8** Melanocyte from 7-day culture which was washed free of medium and stabilized in PBS for 1 h before exposure to $10^{-3}$ M 4-OHA plus tyrosinase 15 $\mu g\, ml^{-1}$ for 1 h. The cell exhibits damage similar to that suffered by the melanocyte in Figure 5, but with more extensive vacuolization of the cytoplasm. This order of damage is classed as severe (3+). ×12,000.

Melanocytes of cultures washed free of medium and stabilized for 1 h in PBS were unaffected by exposure to $10^{-3}$ M 4-OHA for 1 h. Addition of tyrosinase 15 $\mu g\, ml^{-1}$ with $10^{-3}$ M 4-OHA to these washed cultures resulted in severe damage to melanocytes within 1 h, similar to or even greater than that suffered by unwashed cells exposed to $5 \times 10^{-3}$ M 4-OHA plus tyrosinase for 6 h (compare Figures 5 and 8). Very severe damage resulted from exposure of melanocytes for 6 h to $10^{-3}$ M 4-OHA plus tyrosinase. Non-keratinized keratocytes of washed cultures were also damaged, though in general not to the same degree as melanocytes (Figure 9).

The appearances described and illustrated for different drug concentrations and exposure times applied to practically all of the two cell types concerned present in cultures. Partially or fully keratinized keratocytes were, in general, not appreciably affected as far as could be judged from their morphological appearances.

A summary of results is given in Table I.

*Reaction of 4-OHA with tyrosinase without culture medium*

The present investigations confirmed and extended Riley's (1969) previous observations on 4-OHA as substrate for tyrosinase. As shown in Figure 10, 4-OHA diminishes with time and a product formed by the tyrosinase-mediated oxidation of 4-OHA, with maxima of absorbance at 255 and 413 nm, rose to the maximum within 1 h. This product has been identified by Passi & Nazzaro-Porro (1981) as the o-quinone of 3-4-dihydroxyanisole (4-OHA quinone). It began to diminish after 1 h, while new compounds, representing intermediate metabolic products leading to the formation of a 4-OHA-

**Figure 9** Non-keratinized keratocytes of 7-day culture which was washed free of medium and stabilized in PBS for 1 h before exposure to $10^{-3}$ M 4-OHA plus tyrosinase 15 $\mu$g ml$^{-1}$ for 1 h. Note in comparison with control Figure 4, loss of cytoplasmic substance and organelles. This order of damage is classed as severe (3+). ×10,000.

dependent melanoid polymer appeared (Figure 10). After 3 h, merely traces of the 4-OHA quinone were detectable. This fact could indicate, according to data on the tyrosinase-mediated oxidation of dopa (Tomita *et al.*, 1980), that tyrosinase was inactivated, and in fact, further addition of tyrosinase at 3 h gave rise to a new consumption of 4-OHA, and to further formation of new 4-OHA quinone.

The quantity of 4-OHA metabolized by tyrosinase varied with the 4-OHA concentration and with time, as is possible to see from the results in Table II, and from Figure 11, indicating the absolute amounts of 4-OHA removed at shorter times. From Table II it is also evident that 4-OHA continued to be consumed up to and beyond the 24th hour, notwithstanding the fact that the tyrosinase was inactivated.

**Table II** 4-OHA consumption (%) in presence of tyrosinase (15 $\mu$g ml$^{-1}$) without culture medium at different times

| *4-OHA Concentration (M)* | *Time* 12 *min* | 30 *min* | 60 *min* | 90 *min* | 3 *h* | 24 *h* | 48 *h* |
|---|---|---|---|---|---|---|---|
| | *4-OHA consumption* (%) | | | | | | |
| $10^{-4}$ | 60 | 83 | 90 | 95 | 100 | | |
| $5\times10^{-4}$ | 43 | 60 | 74 | 86 | 100 | | |
| $10^{-3}$ | 32 | 46 | 55 | 77 | 98 | 100 | |
| $2\times10^{-3}$ | 18 | 23 | 26 | 31 | 50 | 81 | 96 |
| $5\times10^{-3}$ | 13 | 16 | 21 | 28 | 33 | 60 | 82 |
| $10^{-2}$ | 10 | 11 | 12 | 24 | 27 | 35 | 43 |

Analyses were performed by RP-HPLC. For details see text.

*Reaction of* 4-*OHA with tyrosinase in the presence of culture medium*

For this investigation, due to the great number of UV-absorbing substances present in the culture medium, it was necessary to use, instead of the Liquid Chromatograph, the less sophisticated scanning spectrophotometric method, which does not allow quantification of 4-OHA consumption.

In a control system with 4-OHA and culture medium without tyrosinase, at all concentrations to which the cells were exposed, 4-OHA remained unaltered up to 24 h.

In a system containing 4-OHA, tyrosinase, and culture medium, as shown in Figure 11, 4-OHA again acted as substrate for tyrosinase. However, in contrast to the situation where the reaction took place in the absence of culture medium, the 4-OHA quinone disappeared just after its formation, while one or more new compounds with maximum of absorbance at 485–490 nm were rapidly formed and increased with time up 24 h and more. Their maximum of absorbance and increase with time indicate that these products were completely different from the metabolic compounds normally formed by the reaction between 4-OHA and tyrosinase.

### Discussion

The observations presented here extend those of a previous ultrastructural study (Breathnach *et al.*, 1981) which failed to demonstrate a specific tyrosinase-dependent cytotoxic effect of 4-OHA on

**Figure 10** 4-OHA—tyrosinase reaction without culture medium. RP-HPLC separation of 4-OHA and its catabolites. 4-OHA: $10^{-2}$ M; tyrosinase: 15 $\mu$g ml$^{-1}$. A, B, C, D, E: 4-OHA—tyrosinase reaction at different times. A = 0; B = 12 min; C = 30 min; D = 90 min; E = 3 h. Peaks: 1 = 4-OHA; 2 = 4-OHA quinone.

*Peak areas* A, 1: 475,000. B, 1: 427,500; 2: 12,400. C, 1: 422,750; 2: 28,300. D, 1: 361,000; 2: 23,400. E, 1: 346,750.

The other peaks in the figure represent the intermediate metabolic products leading to the melanoid polymer. They were not identified and peak areas were not measured.

normal human melanocytes in tissue culture such as has been reported for guinea-pig melanocytes (Riley, 1970; Riley *et al.*, 1975). This lack of effect on the human melanocytes could have been attributed to a low level of tyrosinase within the cells and, in an attempt to overcome this, tyrosinase as well as 4-OHA was this time added to the culture medium. This seemed a reasonable approach in view of reports that such a measure had a marked effect on the sensitivity of Harding-Passey melanoma cells to 4-OHA (Dewey *et al.*, 1977). In the event, no difference was observed when tyrosinase was combined with a concentration of 4-OHA ($10^{-3}$ M) which alone, after 30 min, was reported to have a marked cytotoxic effect on guinea-pig melanocytes. Damage to melanocytes was only observed with tyrosinase and significantly higher concentrations of 4-OHA ($5\times10^{-3}$ M, and $10^{-2}$ M) applied to the cultures for longer periods (6–24 h). This damage can in no way be regarded as *specific* since exactly similar damage was suffered by keratocytes in the same circumstances. The damage cannot be attributed to addition of tyrosinase *per se* to the medium, since controls with tyrosinase alone revealed no damage to either cell type.

Because of the continuing discrepancy between our results with normal human melanocytes and those of other authors with animal and tumour cells, the metabolic pathway of the 4-OHA-tyrosinase reaction was examined both in the presence and the absence of culture medium. With a system without culture medium we confirmed and extended previous results (Riley, 1967; Passi & Nazzaro-Porro, 1981), showing that 4-OHA is a substrate for tyrosinase (Figure 10). The tyrosinase-mediated oxidation of 4-OHA gave rise to a 4-OHA quinone which reached maximum concentration within 1 h. That this substance is indeed a quinone—a class of compounds whose toxicity in general is well known—is very likely because of its maxima of absorbance and reactivity (Riley, 1969; Passi & Nazzaro-Porro, 1981). This 4-OHA quinone diminished over a further period of 2 h, while new compounds leading to the formation of the 4-OHA dependent melanoid polymer began to appear and increased with time (Figure 10).

**Figure 11** Absolute amounts of 4-OHA removed at the shorter times (up to 3 h). Each point was calculated on the basis of data contained in Table II.

Many authors believe (Sealey *et al.*, 1980) that the polymerization is essentially random and partly depending upon the constituents of the environment. If this be so, then it is likely that the following are involved in the cyclization leading to the final polymeric product: (a) 4-OHA, the 4-OHA quinone, and the intermediate formed between them; (b) nucleophiles in the environment, including 4-OHA itself, which interact with 4-OHA quinone. This might explain the further consumption with time of 4-OHA, notwithstanding inactivation of the enzyme (Table II).

That nucleophilic substances easily react with quinones, which are strong oxidising and extremely reactive agents, is well known. For example, Dawson & Tarpley (1963) demonstrated that during oxidation of cathecol by tyrosinase, the formation of one of the first products of cyclization, i.e. tetrahydroxydiphenil, involves a nucleophilic attack of cathecol itself on the o-benzoquinone which is the first product of the oxidation. According to them, hydroxide ions and buffer anions may also behave as nucleophiles during the polymerization; such substances were present in our culture media.

Failure of a previous attempt (Breathnach *et al.*, 1981) to produce damage to cultured melanocytes by exposure to 4-OHA alone can be partly explained by the present results. The damage observed in present cultures washed free of medium and exposed to $10^{-3}$ M 4-OHA plus tyrosinase for 1 h, confirms the biochemical findings of production of a free toxic 4-OHA quinone during this period. The absence of such early damage at concentrations of 4-OHA up to $5 \times 10^{-3}$ M in the presence of medium, is due to the fact that the toxic 4-OHA quinone reacted with nucleophilic substances contained in the medium, being thereby inactivated.

**Figure 12** 4-OHA reaction in presence of culture medium. Oxidation of $10^{-2}$ M 4-OHA by tyrosinase (15 $\mu$g ml$^{-1}$) at 25°C at different times: 1 min: 4-OHA quinone (maximum of absorbance 413 nm). At 5 min the 4-OHA quinone disappeared while one or more new compounds with maximum of absorbance at 485–490 nm were formed and increased with time (5 min; 12 min; 30 min; 1 h; 6 h). At 12 h, 24 h, and 48 h, it was necessary to dilute the starting material. Maxima of absorbance remained the same after dilution (485–490 nm).

The later (after 6 h) damage to both keratocytes and melanocytes could be due to the newly formed substances resulting from these reactions (Figure 12). Although the present results clearly show that normal human melanocytes can be damaged by external exposure to products of the oxidation of 4-OHA by tyrosinase, they still do not account for the discrepancy between results on human and guinea-pig melanocytes exposed to 4-OHA alone (Riley, 1970). The possibilities discussed previously (Breathnach *et al.*, 1981) still remain.

One further point of interest emerges from the present investigations. In every instance in which damage to melanocytes occurred, similar damage was suffered by keratocytes. This might be explained in the light of Riley's (1969) demonstration of the capability of 4-OHA to inhibit isolated mitochondrial respiration and protein synthesis, a possibility that could be tested by investigating its effect on cultures of other cell types. The present findings underline the necessity when assessing the effects of potentially cytotoxic drugs on melanocytes, to bear in mind possible similar effects on keratocytes.

This work was supported by grants from The Cancer Research Campaign, the Wellcome Trust, Schering AG, Berlin, and by Grant No. 800160096, control of tumour growth, C.N.R. Italy.

**References**

BREATHNACH, A.S., DIALA, E.B., GALLAGHER, S.M., NAZZARO-PORRO, M. & PASSI, S. (1981). Ultrastructural observations on the effect of 4-hydroxyanisole on normal human melanocytes in tissue culture. *J. Invest. Dermatol.*, **77**, 292.

DAWSON, R.C. & TARPLEY, W.B. (1963). On the pathway of the cathecol-tyrosinase reaction. *Ann. N.Y. Acad. Sci.*, **100**, 937.

DEWEY, D.L., BUTCHER, F.W. & GALPINE, A.R. (1977). Hydroxyanisole induced regression of the Harding-Passey melanoma in mice. *J. Pathol.*, **122**, 117.

EISINGER, M. & MARKO, O. (1982). Selective proliferation of normal human melanocytes *in vitro* in the presence of phorbal ester and cholera toxin. *Proc. Natl Acad. Sci.*, **79**, 2018.

FITZPATRICK, T.B. & BREATHNACH, A.S. (1963). Das epidermale Melanin-Einheit-System. *Dermatol. Wochenschr.*, **147**, 481.

FRITSCH, P., TAPPEINER, G., POHLIN, G. & SCHULER, G. (1981). The culture of normal mammalian melanocytes. In: *The Epidermis in Disease*. (Eds. Marks & Christophers) Lancaster: MTP Press Ltd., p. 459.

GALPINE, A.R. (1981). Ph.D. thesis, University of London.

MORGAN, B.D.J., O'NEILL, J., DEWEY, D.L., GALPINE, A.R. & RILEY, P.A. (1981). Treatment of malignant melanoma by intravascular 4-hydroxyanisole. *Clin. Oncol.*, **7**, 227.

PASSI, S. & NAZZARO-PORRO, M. (1981). Molecular basis of substrate and inhibitory specificity of tyrosinase: phenolic compounds. *Br. J. Dermatol.*, **104**, 659.

RILEY, P.A. (1969). Hydroxyanisole depigmentation: *In vitro* studies. *J. Pathol.*, **97**, 193.

RILEY, P.A. (1970). Mechanism of pigment cell toxicity produced by hydroxyanisole. *J. Pathol.*, **101**, 163.

RILEY, P.A., SAWYER, B. & WOLFF, M.A. (1975). Melanocytotoxic action of 4-hydroxyanisole. *J. Invest. Dermatol.*, **64**, 86.

SEALEY, R.C., FELIX, C.C., HYDE, J.S. & SWARTZ, H.M. (1980). Structure and activity of melanins. In: *Free Radicals in Biology*, Vol. IV. (Ed. Prior) New York: Academic Press, p. 209.

TOMITA, Y., HARIN, A., MIZUMO, C. & SEIJI, M. (1980). Inactivation of tyrosinase by Dopa. *Proceedings of XIth International Pigment Cell Conference*, Sendai, p. 27.